# In-Depth Performance Analysis and Comparison of Monolithic and Particulate Zwitterionic Hydrophilic Interaction Liquid Chromatography Polymer Columns

**DOI:** 10.3390/molecules28072902

**Published:** 2023-03-23

**Authors:** Haibin Li, Zhengjin Jiang, Gert Desmet, Deirdre Cabooter

**Affiliations:** 1 Department for Pharmaceutical and Pharmacological Sciences, Pharmaceutical Analysis, University of Leuven (KU Leuven), Herestraat 49, 3000 Leuven, Belgium; 2 Institute of Pharmaceutical Analysis, College of Pharmacy, Jinan University, Guangzhou 510632, China; 3 Department of Chemical Engineering, Vrije Universiteit Brussel, Pleinlaan 2, 1050 Brussel, Belgium

**Keywords:** HILIC, band broadening, kinetic plot, characteristic length, molecular weight

## Abstract

The kinetic performance of different zwitterionic hydrophilic interaction liquid chromatography polymer columns is evaluated and compared in-depth. For this purpose, two lab-made monolithic columns, synthesized with different crosslinkers, and a commercial particle packed column are considered. It is found that performance evaluation techniques, such as comparing plate height curves or fitted A-, B- and C-terms, obtained by fitting experimental plate height data to a plate height model, are complicated by the determination of a reliable characteristic length. This is due to the very different morphology of these column types, and the heterogeneity of the monolithic columns. The occurrence of a convective flow through the packed particle column further complicates the interpretation of the obtained fitting parameters, as part of the C-term is wrongfully attributed to the A-term. Therefore, the use of the kinetic plot method is suggested for the comparative evaluation of these columns, as kinetic plots do not require the determination of a characteristic length, nor rely on any fitting parameters. With the kinetic plot method, it is demonstrated that the lab-made monolithic columns outperform the packed particle column for plate counts between 10,000 and 800,000. This is attributed to the higher column efficiency of these columns, due to their small domain and skeleton sizes, and their high permeability, resulting from their high external porosity and the occasional occurrence of preferential flow paths.

## 1. Introduction

Modern liquid chromatography techniques have recently gained a lot of popularity due to their extended separation capabilities for complex samples [1]. Among these, hydrophilic interaction liquid chromatography (HILIC) has been used for the analysis of polar compounds, such as metabolites and degradation products [2,3], carbohydrates and aminoglycosides [4,5,6], amino acids, peptides and proteins [7,8,9], for which reversed-phase liquid chromatography (RPLC) is largely inadequate. This popularity of HILIC is in part related to the introduction of commercially available stationary phases with a broad variety of chemistries that allow an adequate tuning of the separation selectivity. In HILIC, the mobile phase typically consists of a large amount of organic solvent, such as acetonitrile (ACN), to which a small amount of water or aqueous buffer is added. The polar stationary phase will absorb water from the mobile phase to form a water-rich layer at its surface. Analytes can then partition from the mobile phase into this water-rich layer for retention [10,11]. Other retention mechanisms such as ionic, adsorption and hydrophobic interactions are also possible, depending on the analyte, the stationary phase type and the mobile phase [12,13].

Examples of typical HILIC stationary phases are bare silica, amide, amino, cyano, diol and zwitterionic phases [12,13]. Zwitterionic stationary phases carry both positively and negatively charged functional groups, such as sulfoalkylbetaine or amino-phosphate functional groups, attached to a suitable backbone. In accordance with recent developments in RPLC, HILIC stationary phase carriers have undergone a similar evolution, with the recent introduction of small (sub-2 µm) particle packed columns, core–shell particles and monolithic structures [14,15,16]. HILIC stationary phases can, moreover, be silica or polymer-bonded, where polymer-bonded columns can be used over a larger pH-range with respect to silica-bonded columns. An often-cited downside of polymer columns is their low mass transfer for small molecules, due to their low proportion of mesopores, severely restricting diffusion. 

Whereas several studies have been devoted to the fundamental comparison of the kinetic performance of particulate and monolithic silica columns, much fewer studies have compared the kinetic performance of their polymer counterparts. Customarily, band broadening in a packed (monolithic or particulate) bed can be described by the general plate height model [17,18,19,20]:

(1) $$\begin{array}{l} H=H_{inhom}+2\frac{D_{eff}}{u_{i}}\left(1+k^{\prime \prime }\right)+\frac{2}{\alpha }\frac{{k^{\prime \prime }}^{2}}{{\left(1+k^{\prime \prime }\right)}^{2}}\frac{\varepsilon_{e}}{1-\varepsilon_{e}}\frac{d^{2}\;u_{i}}{Sh_{m}\;D_{m}}\\ \;\;\;\;\;\;\;\;\;\;\;\;\;\;\;\;\;\;\;\;\;\;\;\;+\frac{2}{\alpha }\frac{k^{\prime \prime }}{{\left(1+k^{\prime \prime }\right)}^{2}}\frac{d^{2}\;u_{i}}{Sh_{pz}D_{pz}} \end{array}$$

With H the plate height; u_i_ the interstitial velocity; D_eff_, D_pz_ and D_m_ the effective, porous zone, and bulk molecular diffusion coefficients, respectively; k″ the zone retention factor; ε_e_ the external porosity; α a geometrical constant; Sh_m_ and Sh_pz_ the Sherwood numbers relating to the mobile zone and the porous zone, respectively, and d a characteristic length. In Equation (1), the first term ($H_{inhom}$) relates to band broadening originating from flow heterogeneities in the bed (traditionally referred to as eddy dispersion). The second term (*B*-term) represents the effective longitudinal diffusion. The third and fourth terms (*C_m_*-, and *C_s_*-terms) are the resistance to mass transfer in the mobile and stationary zones, respectively. The zone retention factor is calculated as:

(2) $$k^{\prime \prime }=\frac{t_{R}\cdot u_{i}}{L}-1$$

With t_R_ the analyte retention time, L the column length and u_i_ the interstitial velocity:

(3) $$u_{i}=\frac{F}{\varepsilon_{e}\pi r^{2}}\;$$

With F the flow rate and r the radius of the column. 

For particle packed columns, the characteristic length d is typically equal to the particle size d_p_, while for monolithic columns, several measures can be used, such as the domain size (d_dom_), the throughpore size (d_tp_) and the skeleton size (d_skel_). Note that:

(4) $$d_{dom}=d_{tp}+d_{skel}$$

In the present study, the performance of two monolithic zwitterionic polymer HILIC columns (poly(SPE-co-EDMA) and poly(SPE-co-MBA) monolithic column, respectively) is compared with that of a packed particle zwitterionic polymer HILIC column (ZIC-pHILIC). Previous work demonstrated that these columns display very low B-term coefficients (or, equivalently, very low effective diffusion coefficients D_eff_) and intra-particle diffusion coefficients (D_pz_, where “pz” stands for “mesoPorous Zone”), which was attributed to very low surface diffusion rates, a strongly hindered diffusion in the polymer backbone, slow localized adsorption events, or a combination thereof [21]. 

The present study aims to further look into the effect of these very low diffusion coefficients on the overall performance of the two column formats and compare and analyze their kinetic performance in more detail.

## 2. Results and Discussion

### 2.1. Geometrical Characterization

**Figure 1:**
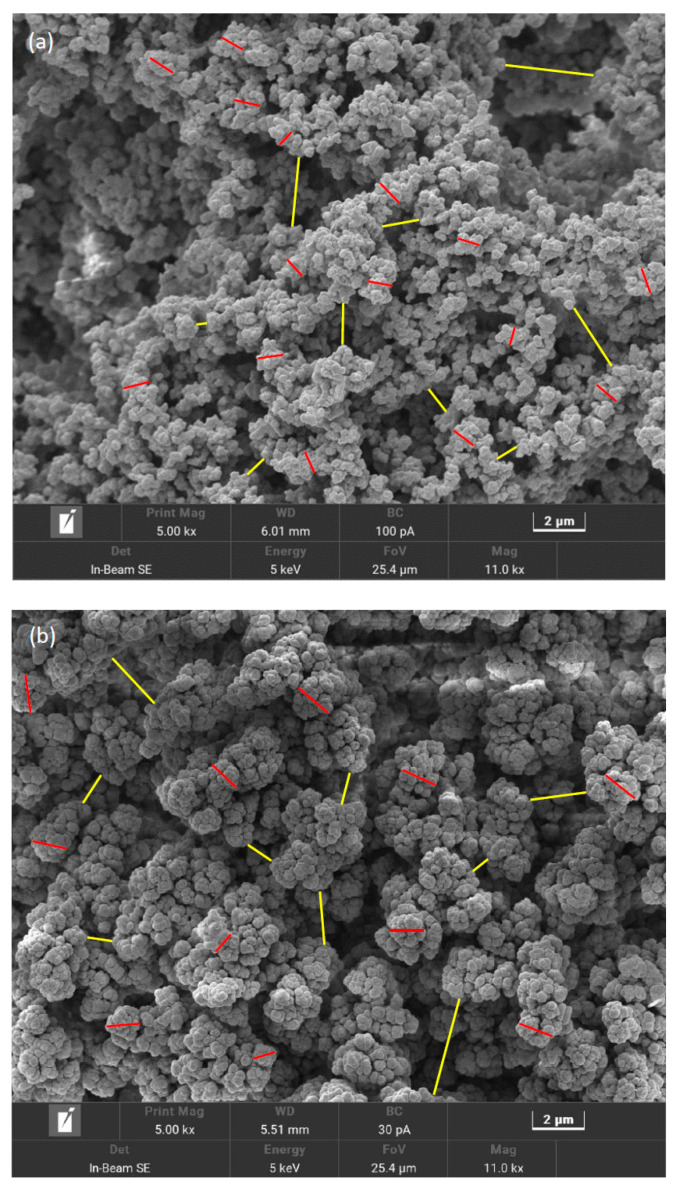

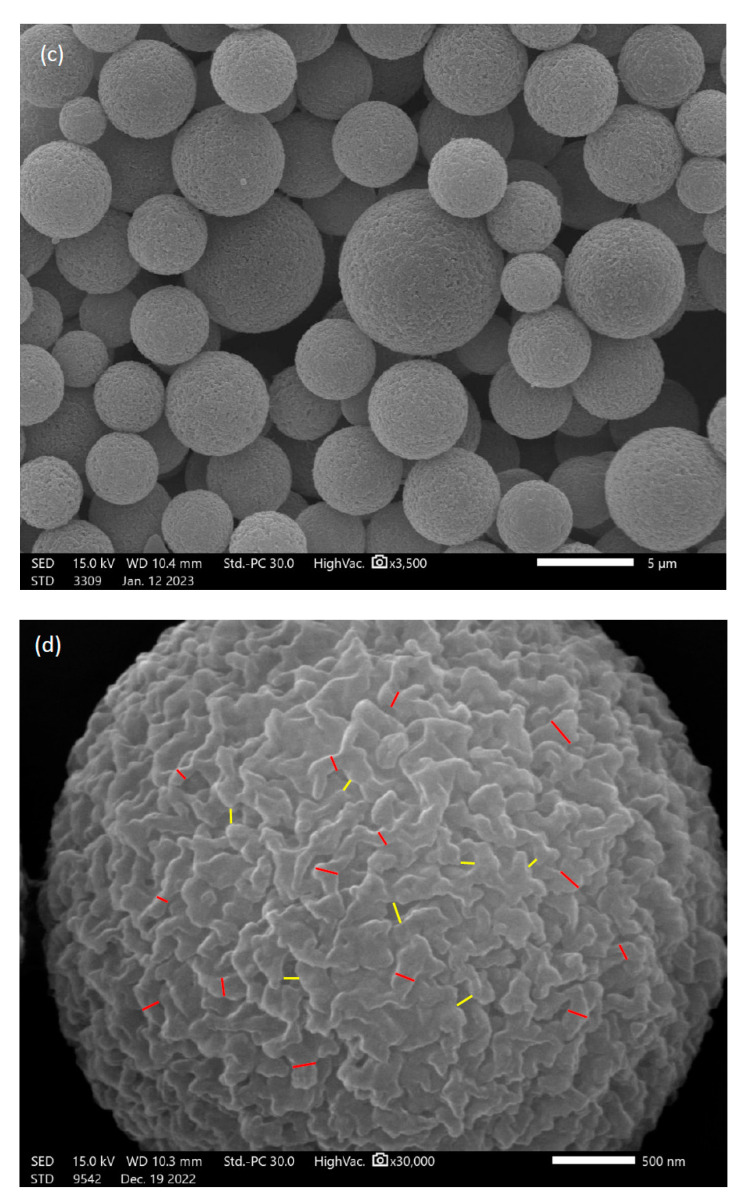
Scanning electron microscopy (SEM) pictures of the evaluated column materials. (**a**) poly(SPE-co-EDMA) monolithic stationary phase, (**b**) poly(SPE-co-MBA) monolithic stationary phase, (**c**) ZIC-pHILIC particles and (**d**) close-up of particle surface. Red lines in (**a**,**b**) indicate the size of d_glob_ and yellow lines the size of d_tp_. Red lines in (**d**) indicate the ‘skeleton’ sizes of the polymer structure, yellow lines indicate the mesopore sizes.

SEM images obtained for the different columns are shown in Figure 1. Both monolithic columns exhibited uniform spherical microglobules agglomerated into larger structures. The microglobules of the poly(SPE-co-EDMA) monolithic column (Figure 1a) were smaller and seemingly had more voids between them compared to the poly(SPE-co-MBA) monolithic column (Figure 1b). The latter exhibited a more crowded, cauliflower-type morphology, that visually seemed more homogeneous compared to the poly(SPE-co-EDMA) column. Additionally, both monolith types displayed a large number of oversized voids, that were randomly positioned. It is not entirely clear whether these voids were inherently present in the monolithic structures, or whether they were a consequence of their preparation for taking the SEM pictures, in which case a piece of 0.5 cm was cut off each monolith. The domain size (d_dom_) of the monoliths was determined from the skeleton size (d_skel_) and the throughpore size (d_tp_), according to Equation (4), by analyzing at least 100 of each for each monolith, as indicated in Figure 1. Note that for d_skel_, intermediate-sized, repeating units were considered, while for the determination of d_tp_, the largest voids were omitted (as can for example be observed on the left side of Figure 1a). The average d_skel_ and d_tp_ of the poly(SPE-co-EDMA) monolithic column were in this way estimated to be 0.7 µm and 0.8 µm, respectively, resulting in a domain size of 1.5 µm, while the skeleton size and throughpore size of the poly(SPE-co-MBA) monolithic column were determined to be 1.7 µm and 1.6 µm, respectively, resulting in a domain size of 3.3 µm, reflecting the smaller features of the poly(SPE-co-EDMA) monolithic column. These values are shown in Table 1, together with the standard deviations (SD) for at least 100 measurements. It should be noted that the SD are generally high, especially for the throughpore sizes (coefficient of variation ~50%), demonstrating the high heterogeneity of the throughpores. 

Figure 1c shows a representative SEM picture obtained for the ZIC-pHILIC particles, indicating the spherical shape of the particles. From Figure 1c it can, however, also be observed that the size of the particles varies quite significantly. The analysis of 200 particles resulted in a mean number-based particle size of 4.7 ± 0.8 µm, in line with the particle size stated by the manufacturer (5 µm). The mean volume-based particle size was 5.3 ± 1.2 µm. Figure 1d shows an enhanced magnification of the surface of one particle. From this picture, the polymer structure of the particle is clearly visible, revealing an internal monolith-like structure with skeletons and throughpores. Although no information about the mesopore size of these particles could be retrieved from the manufacturer, visual analysis of the SEM pictures suggests a skeleton size of 0.13 ± 0.05 µm and a mesopore size of 0.10 ± 0.04 µm.

External porosity values ε_e_ for each column were determined in [21] via ISEC experiments, while total porosities ε_T_ were determined from the elution time of toluene using tetrahydrofuran as the mobile phase. The porosity of the porous zone ε_pz_ was calculated as:

(5) $$\varepsilon_{pz}=\frac{\varepsilon_{T}-\varepsilon_{e}}{1-\varepsilon_{e}}\;$$

The resulting values are also shown in Table 1. 

Both the poly(SPE-co-EDMA) and poly(SPE-co-MBA) monolithic columns displayed very similar ε_e_-values of around 69%, in line with typical ε_e_-values observed for monolithic columns. The poly(SPE-co-MBA) monolithic column, however, had a smaller total porosity $\varepsilon_{T}$, implying a significantly smaller internal porosity of ε_pz_ =12.5% compared to the ε_pz_ = 29.5% for the poly(SPE-co-EDMA) column. 

For the ZIC-pHILIC column, a total porosity of around 60% was obtained, in line with typical ε_e_-values for particle packed columns. The external porosity as measured via ISEC, however, was rather large (around 44%), especially considering the relatively large particle size distribution that was deduced from the SEM pictures. Whereas most random packings of spherical particles have external porosities of 36–40%, packings consisting of particles with a higher particle size distribution are known to yield smaller ε_e_-values since the smaller particles can position themselves between the larger particles and in this way fill up some of the ‘gaps’ in the interstitial volume. In fact, in [22] it was demonstrated that dense random packings of particles with a mean sphericity of 0.86 and a dimensionless standard deviation between 0.1 and 0.6, have external porosities ranging between 35% and 30%, respectively. For a dimensionless standard deviation of 0.23, as is the case for the ZIC-pHILIC particles considered here, ε_e_ would be 34%. Assuming the ISEC measurements are inaccurate because of the large intra-particle voids visible in Figure 1d, we therefore considered a value of 34% for the external porosity ε_e_ and a value of 40% for ε_pz_ for the ZIC-pHILIC column in what follows.

### 2.2. Evaluation of Column Performance

#### 2.2.1. Plate Height Curves

**Figure 2:**
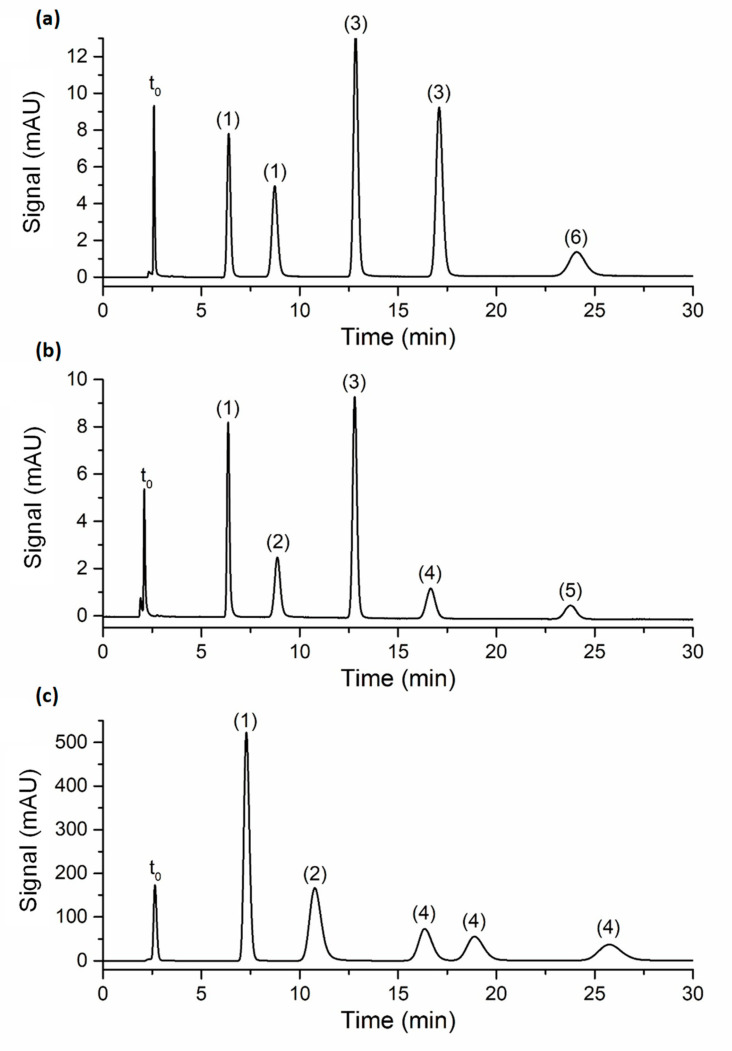
Chromatograms obtained on the (**a**) poly(SPE-co-EDMA) monolithic column, flow rate = 0.0006 mL/min; (**b**) poly(SPE-co-MBA) monolithic column, flow rate = 0.0006 mL/min; (**c**) ZIC-pHILIC column, flow rate = 0.1 mL/min. Mobile phase compositions are shown in Table S1; column temperature: room temperature; peak annotation: (1) uracil, (2) adenosine, (3) thiourea, (4) uridine, (5) inosine, (6) hypoxanthine. The t_0_-marker was toluene.

The efficiency of the poly(SPE-co-EDMA) and poly(SPE-co-MBA) monolithic columns, and the ZIC-pHILIC column was subsequently evaluated using a series of polar compounds with similar characteristics (nucleobases and nucleotides). To ensure similar retention factors on all columns, the composition of the mobile phase was adapted for each compound and column individually, as shown in Table S1 in the Supporting Information. Figure 2 shows representative chromatograms for each column, with zone retention factors varying between k″~2 and k″~10. As can be observed from Figure 2, good peak shapes were obtained on all columns (with tailing factors <1.3), showing that the performance of the lab-made monolithic columns was not disturbed by the occurrence of, for example, excessive preferential flow paths or undesirable dual retention mechanisms.

**Figure 3:**
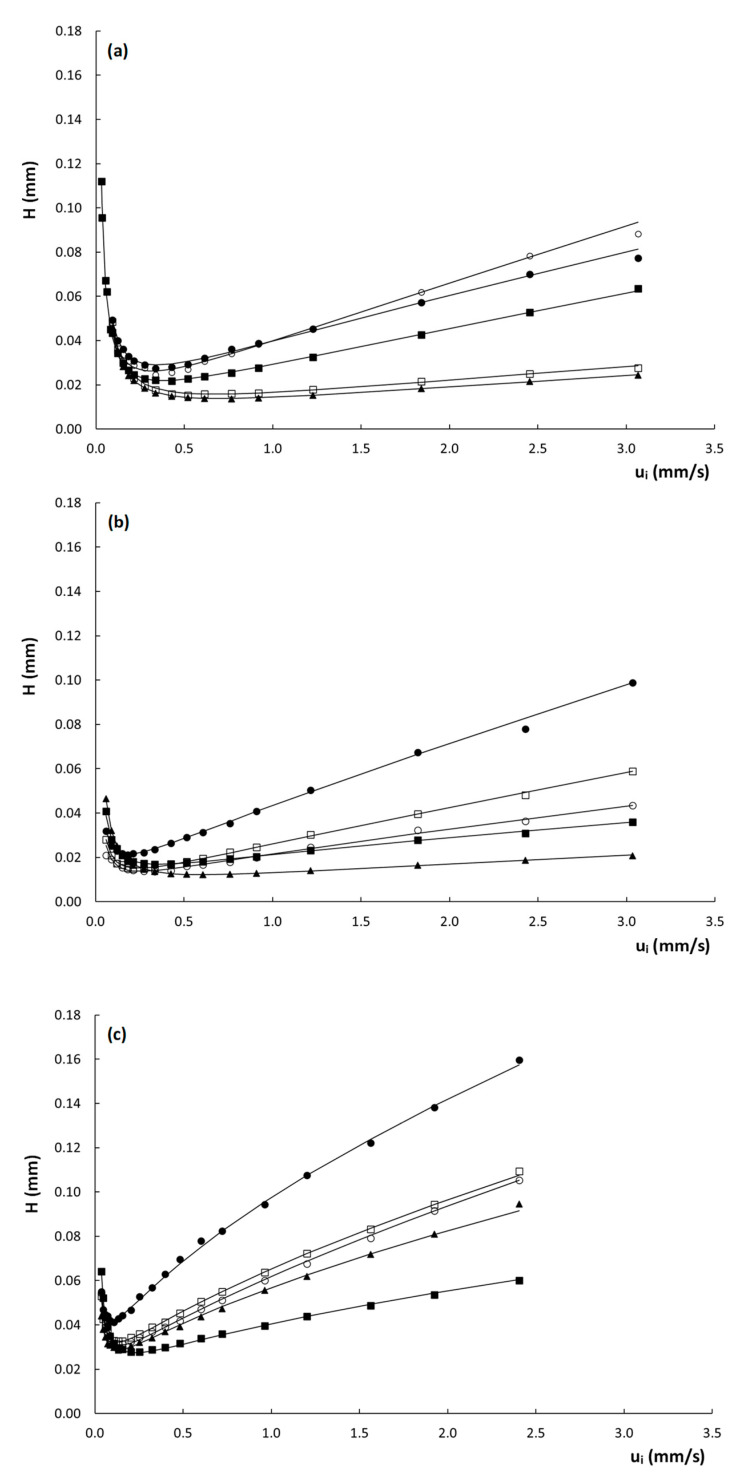
Plate height curves of H versus u_i_ obtained on the (**a**) poly(SPE-co-EDMA) monolithic column, (**b**) poly(SPE-co-MBA) monolithic column and (**c**) ZIC-pHILIC column. k″ = 1.9–2.2 (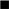), k″ = 2.9–3.7 (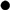), k″ = 4.7–6.4 (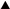), k″ = 6.4–7.3 (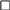), k″ = 9.7–10.5 (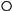). Mobile-phase conditions are given in Table S1.

To evaluate the efficiency of the columns, Figure 3 shows the obtained curves of plate height (H) versus interstitial velocity ($u_{i}$) for the three columns. Note that the interstitial velocity was preferred over the linear velocity for the construction of the plate height curves, as the former is fundamentally more sound, since band broadening essentially occurs because part of the molecules are stagnant in the mesoporous zone, while others are moving in the mobile phase. For band broadening to occur, it does not matter whether the molecules in the mesoporous zone are residing in the stagnant mobile phase or retained on the stationary phase. Since the expression for the interstitial velocity (see Equation (3)) does not distinguish between these two, it is hence more suitable to describe the evolution of band broadening with velocity. 

From Figure 3, it is evident that the plate heights obtained on the poly(SPE-co-EDMA) column (Figure 3a) are slightly higher than those obtained on the poly(SPE-co-MBA) column (Figure 3b), despite the smaller characteristic lengths of the former (Table 1). This could be related to the seemingly more homogeneous structure of the poly(SPE-co-MBA) column (Figure 1b). The plate height curves obtained on the particulate ZIC-pHILIC column (Figure 3c) clearly display both higher plate heights and a steeper C-term compared to the monolithic columns. The particulate column also displays a certain degree of flattening or curvature of the plate height curve in the high flow velocity range. Given the monolithic-like structure of the particles (cf. Figure 1d) and the high intra-particle porosity (ε_pz_ = 40%), this flattening of the plate height curve at high velocities could be due to a convective (perfusion) flow through the particles.

To verify this, the ratio of the pore velocity u_pore_ versus the superficial velocity u_s_, with $u_{S}=u_{i}\;\varepsilon_{e}$, was calculated using the correlation developed by Afeyan et al. [23]:

(6) $$\frac{u_{pore}}{u_{S}}=\frac{K_{p}}{K}{\left(\frac{\tau d_{pore}}{d_{p}}\right)}^{2}\frac{\left(1-\varepsilon_{e}\right)}{\varepsilon_{pz}}\;$$

With K_p_ the particle permeability, K the column permeability, d_p_ the particle size and d_pore_ the mesopore size, here taken as equal to 0.10 µm, as deduced from the SEM pictures. 

Note that the permeability K can be estimated as follows:

(7) $$K=\frac{\varepsilon^{3}}{150\;\left(1-\varepsilon \right)²}\;$$

With ε = ε_e_ for K and ε = ε_pz_ for K_p_. The constant τ is related to the tortuosity of the packing, and has a typical value of 2. In this way, it can be calculated that the ratio u_pore_/u_s_ is 0.003, or that 0.3% of the average velocity through the column passes through the mesopores of the particles. Although this seemingly only represents a small percentage of the average velocity, it is also important to consider the ‘time of transport’ through the particles via diffusion versus convection. This can be calculated as:

(8) $$time_{convection}=\frac{d_{p}}{u_{pore}}=0.67\;s\;$$

(9) $$time_{diffusion}=\frac{{d^{2}}_{p}}{D_{eff}}=\frac{{d^{2}}_{p}\cdot \tau^{2}}{\varepsilon_{pz}\cdot D_{m}}=0.17\;s\;$$

Note that u_pore_ in Equation (8) corresponds to an interstitial velocity of u_i_= 2.4 mm/s in the packed bed, the maximum u_i_ measured during the plate height experiments. This is because the higher the velocity is, the more convection will become predominant. The calculations in Equations (8) and (9) show that the transport through the particles via diffusion is only four times more rapid than via convection at this highest velocity, indicating that transport via convection is significant in the ZIC-pHILIC particles. It is, however, not entirely clear whether the observed amount of convection is large enough to explain the observed flattening at the high-velocity end of the plate height curve. A possible explanation for this lower-than-expected contribution of the intra-particle convection could be due to the fact that a number of parameters in these calculations are based on estimations (ε_e_, ε_pz_, d_pore_), that could have resulted in a lower accuracy of the obtained results.

The experimentally obtained plate height data were subsequently fitted to the following plate height equation [24]:

(10) $$H=Au_{i}^{\frac{1}{2}}+\frac{B}{u_{i}}+Cu_{i}\;$$

For this purpose, all three coefficients (A, B and C) were either fitted freely or, alternatively, the B-coefficients were fixed to the values obtained via peak parking in [21], and the A- and C-coefficients were subsequently fitted. The resulting values are shown in Table 2. Note that a relatively good agreement is obtained between the B-term values obtained via fitting and peak parking (average deviation of 10%).

From Table 2, it is moreover clear that the A-term values obtained for the packed bed column are higher than those obtained for the monolithic columns, which are much more similar to each other. The C-term values, on the other hand, are more in line for all columns, which is somewhat surprising considering the fact that the high velocity range of the plate height curves in Figure 3 is much steeper for the packed bed column, than for the monolithic columns. The larger A-terms seem to suggest a lower degree of homogeneity for the particle packed column. It must, however, be kept in mind that the fitted A-term values of the ZIC-pHILIC column are influenced by the assumed perfusion and its concomitant flattening of the plate height curve in the high-velocity range. This perfusion helps to suppress the C-term band broadening but part of the latter is inevitably wrongfully attributed to the A-term during the fitting, since the A-term incorporates a certain amount of flattening via the $u_{i}^{0.5}$-term in Equation (10). This most probably leads to an overestimation of the A-term, and consequently an underestimation of the C-term of the ZIC-pHILIC column, making the fitted A- and C-term values reported in Table 2 unreliable for this column. For the monolithic columns, this is not the case, as the plate height curves are perfectly linear with velocity in the heigh velocity range. 

Since the only term that is linearly proportional with u_i_ in Equation (1) is the C_s_-term (the fourth term), considering the Sh_m_-factor in the third term is velocity-dependent [25], it can be assumed that the plate heights obtained on the monoliths in the high-velocity range are C_s_-term dominated. 

#### 2.2.2. Permeability Measurements

During the plate height measurements, column pressures were carefully monitored as a function of the applied flow rate to determine the column permeability values. Figure S1 in the Supporting Information shows the obtained curves of pressure as a function of the linear velocity u_0_ for the three evaluated columns. Note that for the construction of these curves, preference was given to the linear velocity u_0_ over the interstitial velocity, as the total velocity through the column (including the zero-velocity inside the pores) determines the column permeability under actual separation conditions. The experimental pressure values were fitted to a linear equation, the obtained equations and goodness of fit (represented by R²-values) are also shown in Figure S1. In general, the pressures measured on all columns displayed a linear behavior with respect to the applied velocity, since all R² > 0.998. Looking closer at the values, it was, however, observed that the two monolithic columns displayed higher R²-values of 0.9997, whereas the R²-value obtained on the particulate column was slightly lower (0.998). When fitting the experimental pressure values obtained below 20 bar to a linear equation, a similar excellent R²-value of 0.9996 was obtained for the particle packed column. The values obtained above 20 bar deviated from this linearity in an upward manner. This could indicate that at pressures above 20 bar, the particles in the ZIC-pHILIC column become somewhat compressed and deformed, resulting in higher-than-expected pressures. The monolithic columns do not display this behavior, suggesting the monolithic structures are more mechanically stable at higher pressures, and hence inherently more suited as a chromatographic backbone in the case of polymeric stationary phases. Note that the maximum column pressure applied to all columns was below 100 bar. The maximum allowable backpressure specified by the manufacturer is 200 bar for the ZIC-pHILIC column.

Based on these observations, permeability (K_v0_) values were subsequently calculated via Darcy’s law [26]:

(11) $$K_{v0}=\frac{u_{i}\eta L}{\Delta P}\;$$

**Figure 4:**
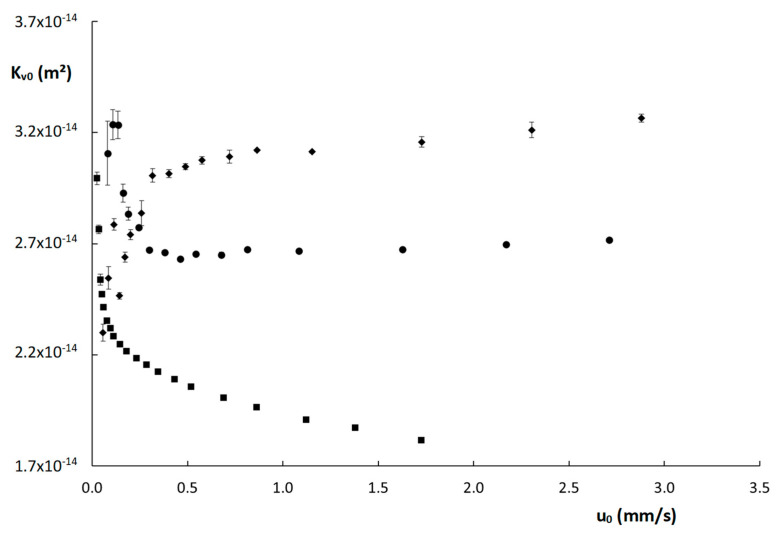
Curves of permeability (K_v0_) as a function of the linear velocity (u_0_) for the columns evaluated in this work: (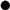) poly(SPE-co-EDMA) monolithic column, (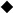) poly(SPE-co-MBA) monolithic column, (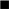) ZIC-pHILIC column. The error bars are the standard deviations obtained from three replicate measurements.

Wherein η is the mobile phase viscosity (Pa·s), L the column length (m) and ΔP is the column pressure (Pa). The resulting K_v0_-values are shown as a function of u_0_ in Figure 4. Interestingly, the K_v0_-values obtained for the two monolithic columns seem to stabilize above u_0_ = 0.5 mm/s, whereas the values obtained for the poly(SPE-co-EDMA) column are higher for smaller u_0_-values, and those obtained on the poly(SPE-co-MBA) column are actually lower. It should be mentioned that the lowest K_v0_-values were obtained below the recommended operational range of the nanoLC flow selector (recommended range 50 nL–1000 nL/min, values measured here starting at 20 nL/min), which could have resulted in these deviating values. Note also that the standard deviations (denoted by the vertical error bars) obtained for the K_v0_-values at these low velocities are clearly larger. However, it is somewhat surprising that both monolithic columns show opposing trends in this low velocity range. For the ZIC-pHILIC column, a generally decreasing trend of K_v0_ with increasing flow rate was observed. This reflects the higher-than-expected observed pressure values and could hence indicate a compression of the packed bed at higher velocities. 

#### 2.2.3. Reduced Plate Height Curves

Since band broadening is not only influenced by differences in uniformity of the evaluated structure (particle packed or monolithic), but also by differences in characteristic length and diffusion properties, reduced plate height curves of h versus ν_i_ were subsequently constructed to further investigate the band broadening behavior observed in the different columns. Note that for the calculation of the reduced plate height h and the reduced interstitial velocity ν_i_ a characteristic length d needs to be specified:

(12) $$h=\frac{H}{d}\;$$

(13) $$\nu_{i}=\frac{u_{i}\cdot d}{D_{m}}\;$$

As mentioned in the introduction, this characteristic length is typically taken equal to the particle size for particle packed columns, while for monolithic columns, the domain size (d_dom_) can for example be used. Figure S2 in the Supporting Information shows the reduced plate height curves that were obtained using the particle size for the particle packed column, and the domain size for the monolithic columns, as specified in Table 1. 

Note that the curves in Figure S2 have all been constructed using the same scale on the x- and y-axis. This representation now shows a completely different picture compared to the curves shown in Figure 3. The reduced plate heights obtained on the ZIC-pHILIC column (h_min_ = 6–9) are now much closer to those obtained on the poly(SPE-co-MBA) column (h_min_ = 4–6), while those obtained on the poly(SPE-co-EDMA) column (h_min_ = 9–18) are much higher. Note also the much steeper slope of the plate height curve in the high velocity range of the latter. This is entirely due to the much smaller characteristic lengths obtained for the poly(SPE-co-EDMA) column (Table 1), impacting the calculation of both the reduced plate height h and the reduced velocity ν_i_, as shown in Equations (12) and (13). However, as was already mentioned earlier, the standard deviations observed for these characteristic lengths were quite large, raising suspicions about the validity of using the domain size as the characteristic length for the monolithic columns. As an alternative, the permeability-based characteristic length proposed by Halasz (d_Halasz_) was therefore investigated next [27]:

(14) $$d_{Halasz}=\sqrt{10^{3}K_{v0}}\;$$

**Figure 5:**
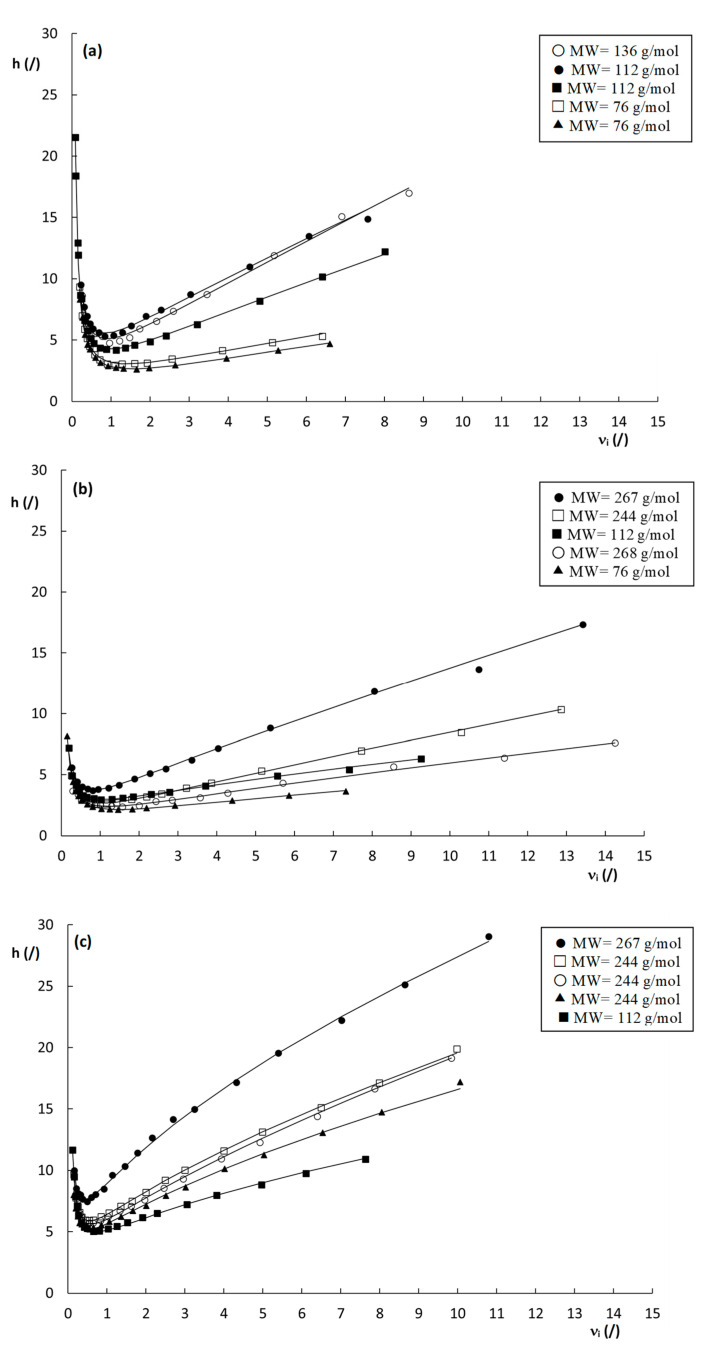
Reduced plate height curves of h versus ν_i_ obtained by using d_Halasz_ (Equation (14)) as the characteristic length for the (**a**) poly(SPE-co-EDMA) monolithic stationary phase, (**b**) poly(SPE-co-MBA) monolithic stationary phase and (**c**) ZIC-pHILIC column. k″= 1.9–2.2 (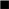), k″= 2.9–3.7 (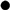), k″= 4.7–6.4 (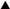), k″= 6.4–7.3 (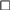), k″= 9.7–10.5 (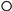). Mobile-phase conditions as in Table S1.

Using the square root of the permeability of a column, the characteristic length is defined in terms of the “price” (pressure drop) that has to be paid for this characteristic length. To calculate d_Halasz_ for the different columns, the permeability values K_v0_ obtained at the highest measured pressure were used. This resulted in values of d_Halasz_ = 5.2, 5.7 and 5.5 µm for the poly(SPE-co-EDMA), the poly(SPE-co-MBA) column and the ZIC-pHILIC column, respectively (Table 1). Note that these values are in very close agreement with each other despite the completely different structure of the packing. Figure 5 shows the reduced plate height curves obtained using d_Halasz_ as the characteristic length. Unsurprisingly, given the close proximity of the d_Halasz_-values, the curves show similar trends as observed for their non-reduced counterparts in Figure 3. Minimum reduced plate heights observed for the monolithic columns are h_min_ = 3–5 for the poly(SPE-co-EDMA) column, and h_min_ = 2–4 for the poly(SPE-co-MBA) column, and hence slightly lower for the latter, in line with the more homogeneous structure of the poly(SPE-co-MBA) column (Figure 1b). Minimum plate heights for the ZIC-pHILIC column are h_min_ = 5–7, and hence larger than for the monolithic column, while also the c-term is steeper and shows the same curvature/flattening as in Figure 3c. Despite the fact that these curves seem to present a more realistic view on the performance of the columns, and are more in line with one another, it should be mentioned that the square root of K_v0_ in fact has no structural meaning, and can hence not be linked to the morphology, disorder or heterogeneity of the columns.

Another interesting observation that can be made from the curves shown in Figure 5, is the seemingly random order of the plate height curves in the high velocity range. In fact, similar random variations of the curve order can also be observed for the plate height curves in Figure 3 and Figure S2, with no obvious link between the steepness of the curves and the corresponding k″–values. Given the high dependency of the plate heights on the c_s_-term (at least for the monolithic columns), which decreases with increasing k″, as can be deduced from Equation (1), this is somewhat surprising as a systematic decrease in the c-term region with k″ would be expected. However, surprisingly, there does seem to be a correlation between the molecular weight (MW) of the compounds (Table S1 in the Supporting Information) and the order of the plate height curves, where the highest MW compounds generally have the steepest c-terms, and the low MW compounds the flattest c-terms. One exception to this behavior is the compound with a k″ = 9.69 on the poly(SPE-co-MBA) column.

To explore these observations further, and assuming the high velocity part of the plate height curve is c_s_-term dominated, at least for the monolithic columns, Equation (1) demonstrates the importance of intra-particle diffusion (D_pz_) in the c_s_-term. D_pz_ can be written as [28]:

(15) $$D_{pz}=\frac{{k_{0}}^{\prime \prime }\gamma_{mp}D_{m}+\left(k^{\prime \prime }-{k_{0}}^{\prime \prime }\right)\gamma_{s}D_{s}}{k^{\prime \prime }}\;$$

In this equation, γ_s_D_s_ represents the stationary phase diffusion, γ_mp_D_m_ the mesopore diffusion, and k_0_″ the zone retention factor k″ for k′= 0. Note that the phase retention factor k’ and the zone retention factor k″ are related as follows:

(16) $$k^{\prime \prime }=\frac{\left(1+k^{\prime }\right)\varepsilon_{T}}{\varepsilon_{e}}-1$$

Substituting k’ = 0 in Equation (16) yields:

(17) $${k_{0}}^{\prime \prime }=\frac{\varepsilon_{T}}{\varepsilon_{e}}-1$$

From Equation (17) it can be understood that k_0_″ is a structural feature of the column packing, depending only on the interstitial porosity ε_e_ and the total porosity ε_T_.

In [21], it was demonstrated that D_pz_/D_m_ and hence D_pz_ are very low for the columns evaluated in this work, and this was attributed to a very low amount of surface diffusion, a very strong and localized adsorption mechanism and/or strongly hindered diffusion in the polymer matrix. Under these circumstances, it can be assumed that the contribution of the stationary phase diffusion to the overall intra-particle diffusion is very low, or in other words:

(18) $$\gamma_{s}D_{s}≅0$$

Substituting Equation (18) in Equation (15), yields:

(19) $$D_{pz}=\frac{{k_{0}}^{\prime \prime }\gamma_{mp}D_{m}}{k^{\prime \prime }}\;$$

From Equations (1) and (19) it then follows that:

(20) $$H_{Cs}=C_{s}\cdot u_{i}=\frac{2}{\alpha }\frac{k^{\prime \prime }}{{\left(1+k^{\prime \prime }\right)}^{2}}\frac{d^{2}\;u_{i}}{Sh_{pz}D_{pz}}=\frac{1}{5\cdot \alpha }\frac{{k^{\prime \prime }}^{2}}{{\left(1+k^{\prime \prime }\right)}^{2}}\frac{d^{2}\;u_{i}}{{k_{0}}^{\prime \prime }\gamma_{mp}D_{m}}\;$$

With α being a geometrical constant (6 for a packed bed column and 4 for a TSM) and Sh_part_ = 10 [17].

Rearranging this leads to an expression for γ_mp_:

(21) $$\gamma_{mp}=\frac{1}{C_{s}}\frac{d^{2}}{5\cdot \alpha }\frac{{k^{\prime \prime }}^{2}}{{\left(1+k^{\prime \prime }\right)}^{2}}\;\frac{1}{\frac{\varepsilon_{T}}{\varepsilon_{e}}-1}\;\frac{1}{D_{m}}\;$$

**Figure 6:**
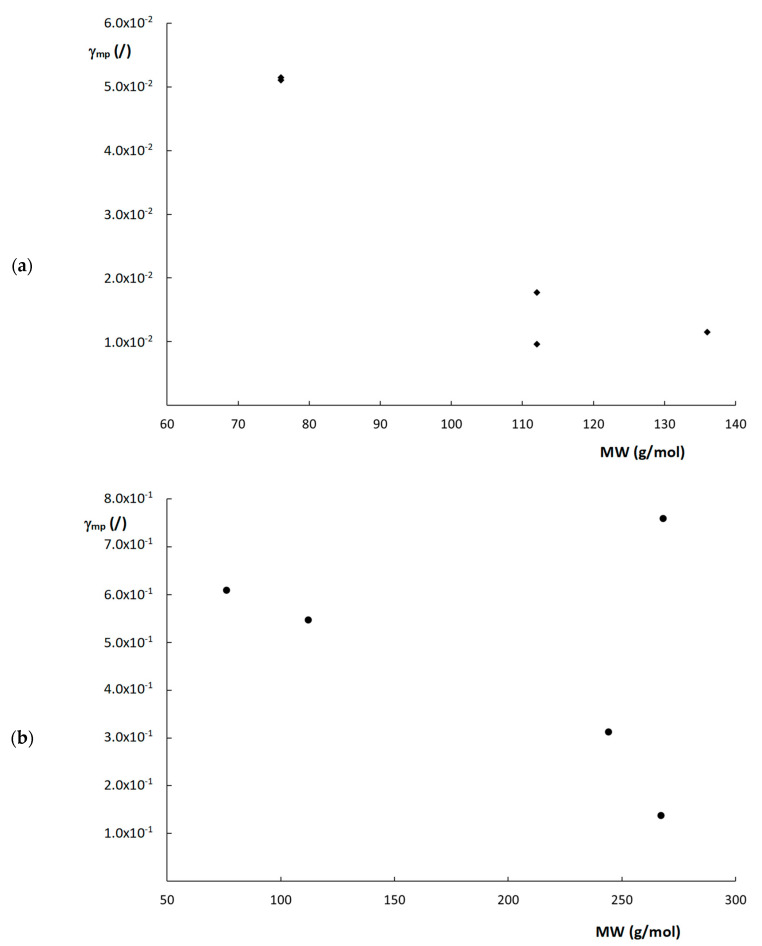
Plots of γ_mp_ versus MW for the (**a**) poly(SPE-co-EDMA) monolithic column and (**b**) poly(SPE-co-MBA) monolithic column.

From Equation (21), it can be deduced that γ_mp_ essentially depends on structural column characteristics (d, ε_e_ and ε_T_), C_s_, D_m_ and k″. Even though the actual value of d is not entirely clear for the monolithic columns, this value is constant per column. In other words, when evaluating a single column, the exact value of d does not matter that much, as long as the same value is consistently used for that column. Therefore, d_dom_ was taken to calculate γ_mp_ for the monolithic columns. C_s_-values were taken as equal to the C-term values obtained by fitting the plate height curves to Equation (10), as shown in Table 2, considering the C-term region was C_s_-dominated. Figure 6 shows the obtained calculated values of γ_mp_ plotted as a function of the MW of the compounds for the two monolithic columns. Interestingly, a rough trend can be observed where γ_mp_ seems to decrease as the MW increases, with the exception of the compound with a MW = 268 g/mol (k″ = 9.69) on the poly(SPE-co-MBA) column. Although this is highly speculative, and more data are required to confirm this trend, these observations seem to suggest that compounds experience more obstruction against free movement in the mesoporous space of the monolithic polymer matrix, as their MW increases. Since it was impossible to obtain the C_s_-term for the ZIC-pHILIC column, as its fitted C-term was also impacted by the observed flattening of the curve, the same calculations were not made for the ZIC-pHILIC column.

#### 2.2.4. Kinetic Plot Analysis

As amply demonstrated in the previous sections, comparing the performance of structurally diverse columns, such as the particle packed column and monoliths investigated in this work, presents a number of difficulties when a suitable characteristic length needs to be selected. For particle packed columns, a good choice seems to be the particle size specified by the manufacturer, although a detailed assessment of the true particle size typically reveals deviating values that can show a certain degree of polydispersity. For monolithic columns, this is even more difficult, as multiple measures can be used, such as the throughpore size, the skeleton size, or the domain size. However, varying degrees of homogeneity throughout the monolithic structure can result in large variations in the obtained values, making it difficult to determine a single representative value. Additionally, it has also been demonstrated that comparing fitted A-, B- and C-term values, as is often done in the literature [14,29,30], can be severely complicated when unexpected phenomena occur, such as perfusion flow through the particles, as this can lead to an incorrect interpretation of the obtained A-, B- and C-term values. 

To remove any uncertainty about the characteristic length when comparing the kinetic performance of structurally diverse columns, and to avoid any complications when interpreting obtained fitting values, an elegant solution to compare column performance is the kinetic plot (KP) method [31,32,33]. Kinetic plots express and compare the performance of columns as the time required to obtain a certain plate count, and are obtained by combining all relevant information of a column in the following equations:

(22) $$t_{0}=\frac{\Delta P_{max}}{\eta }\left[\frac{K_{v0}}{u_{0}^{2}}\right]\;$$

(23) $$N=\frac{\Delta P_{max}}{\eta }\left[\frac{K_{v0}}{u_{0}H}\right]$$

With ΔP_max_ the maximum pressure the column can withstand and η the mobile phase viscosity. 

Note that using Equations (22) and (23), every experimentally obtained datapoint of u_0_ versus H is converted into a measure of the time that is required to obtain a certain plate count when operating the column at the maximum pressure, and at the corresponding velocity u_0_. In this way, each datapoint of t_0_ versus N is in fact obtained in a different column length, where low values of u_0_ will typically be obtained in long column lengths and hence result in high N-values, while high u_0_-values will typically be obtained in short column lengths and hence result in lower N-values. 

**Figure 7:**
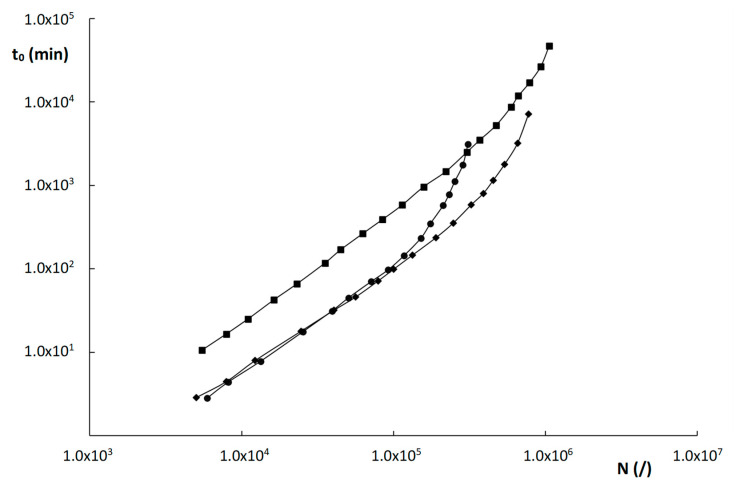
Kinetic plots of time (t_0_) versus plate count (N) for the materials evaluated in this work: (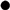) poly(SPE-co-EDMA) monolithic column, (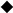) poly(SPE-co-MBA) monolithic column, (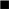) ZIC-pHILIC column. Component = uracil, having k″ ≅ 2 on all three materials.

Kinetic plots for the columns evaluated in this work were constructed for the compound with k″~2 and for a maximum pressure ΔP_max_ = 200 bar and are shown in Figure 7. The compound with k″~2 was the same for all columns (uracil) and was purposely chosen to avoid any bias on the column performance that might be due to MW effects. The plots in Figure 7 show that the two monolithic columns outperform the ZIC-pHILIC column over the entire relevant range of plate counts between 10^3^ and 8 × 10^5^ plates. This is entirely attributed to the higher efficiency of the monolithic columns, as the permeability values of the monoliths and the particle-packed column are relatively similar. 

This higher efficiency of the monoliths is somewhat surprising, given their higher degree of heterogeneity, but seems to be due to the smaller domain and skeleton sizes, that keep their plate heights within check. The relatively high permeability of the monoliths, similar to that of a 5 µm particle-packed column, can in turn be explained by their much higher external porosity and the apparent occurrence of preferential flow paths within the monolithic structure.

The two monolithic columns, moreover, perform similarly well in the range of roughly 5000–120,000 plates. Only for higher plate counts, does the poly(SPE-co-MBA) column outperform the poly(SPE-co-EDMA) column, due to the slightly higher K_v0_-values and slightly better efficiency of the former. The ZIC-pHILIC becomes more performant than the two monolithic columns for very high plate counts (≥10^6^ plates) only. This is due to the slightly lower B-term values that were obtained for the ZIC-pHILIC column, as can be deduced from Table 2. As the high N-range of the kinetic plot is typically obtained at very low velocities, where the B-term is dominant, this lower B-term results in the better performance of the ZIC-pHILIC column in this region.

## 3. Materials and Methods

### 3.1. Reagents and Materials

Adenosine and uracil were obtained from Janssen Chimica (Geel, Belgium). Uridine, thiourea, hypoxanthine and inosine were purchased from Sigma-Aldrich (Steinheim, Germany). Toluene was obtained from Acros Organics (Geel, Belgium).

Glacial acetic acid (99.9% purity) was obtained from Merck (Darmstadt, Germany), ammonium acetate from Sigma-Aldrich. Acetonitrile (ACN) and methanol (MeOH), both HPLC grade, were from Fisher Chemicals (Erembodegem, Belgium). Milli-Q water was prepared in the lab using a Milli-Q system (Millipore, Bedford, MA, USA). 

The following columns were evaluated in this work: a ZIC-pHILIC particle packed column from Merck (2.1 mm I.D. × 150 mm, 5 μm) and two in-house made capillary monolithic columns: a poly(SPE-co-MBA) (0.1 mm I.D. × 226 mm) and a poly(SPE-co-EDMA) column (0.1 mm I.D. × 234 mm). More details on their preparation mode can be found in [34]. Briefly, designated amounts of the hydrophilic monomer (SPE), crosslinker (N,N′-methylenebisacrylamide (MBA) or ethylene dimethacrylate (EDMA)), initiator (AIBN) and porogens (methanol for MBA, water and propanol for EDMA) were accurately weighed and mixed into a 1.5-mL vial. After ultrasonication and degassing for 10 min, the polymerization mixtures were introduced into pre-treated capillaries. Both ends of the capillaries were sealed with GC septa. The capillaries were then submerged into a water bath at 60 °C for 12 h. Finally, the resulting monolithic columns were flushed with methanol overnight in order to remove any residual reagents inside the capillaries. All columns possessed sulfoalkylbetaine zwitterionic functional groups, covalently bonded to the porous polymer beads in the case of the ZIC-pHILIC column. 

### 3.2. Instrumentation

Measurements on the ZIC-pHILIC column were done on an Agilent 1290 UHPLC system (Agilent Technologies, Santa Clara, CA, USA) consisting of a quaternary pump, an autosampler, and a diode array detector with a flow cell of 1 μL. Measurements on the capillary monolithic columns were executed on an Ultimate 3000 RSLC nano system (Dionex, Amsterdam, the Netherlands), with a Binary Rapid Separation Nano Flow pump with nano flow selector, an autosampler, a four-port injection valve with a 20 nL internal loop (VICI, Houston, TX, USA) and a variable wavelength detector (VWD) with a 3 nL flow cell. Experiments were executed at room temperature (21.5 ± 0.5 °C), using an injection volume of 20 nL for the monolithic columns, and 1 μL for the ZIC-pHILIC column. The detection wavelength was set to 254 nm, and the data acquisition rate was 40 Hz for all experiments. Data acquisition and processing were done with Chromeleon software (version 6.8, Dionex) or OpenLab Chemstation software (edition C.01.07, Agilent Technologies).

pH values were measured using a Metrohm 691 pH meter (Antwerp, Belgium). Scanning electron microscopy (SEM) experiments were performed using a TESCAN MIRA4 system (Brno, Czech Republic), using an energy between 5 and 15 keV. Magnifications were between 700× and 50.000×.

### 3.3. Samples and Mobile Phases 

Stock solutions of uracil, thiourea, hypoxanthine, adenosine, uridine and inosine were prepared in a concentration of 2000 ppm in ACN:H_2_O (50:50, *v:v*). These stock solutions were subsequently diluted in pure ACN to a final concentration of 50 ppm for each compound. Mobile phases were prepared by mixing ACN in different ratios with an ammonium acetate solution (adjusted to pH = 6.0 with glacial acetic acid) as shown in Table S1 in the Supporting Information [21]. The ammonium acetate concentration in Table S1 represents the total concentration in the mobile phase. Molecular diffusion coefficients (D_m_) for each compound in their respective mobile phases were determined via Taylor-Aris experiments, as detailed in [21]. The obtained D_m_-values are also given in Table S1.

### 3.4. Plate Height Measurements

Plate heights were measured over a broad range of velocities using the mobile phases shown in Table S1. The composition of the mobile phase was adjusted for each column and compound individually, to obtain similar zone retention factors (k″) at the optimum velocity on each column, ranging between k″ = 2 and k″ = 10. All plate heights were measured in triplicate for at least 16 different velocities on each column. Analyte retention times t_R_ were obtained from the first moments of the peaks, while peak widths were determined at 4.4% of the peak height. All measured data were corrected for the extra-column contribution (ECC). For the Agilent 1290 UHPLC system, the ECC was experimentally determined using a zero-dead volume union instead of the column. For the Dionex Ultimate 3000 RSLC nano system, the ECC was calculated from the geometrical volume of the tubing, the injection volume and the volume of the flow cell. 

## 4. Conclusions

A detailed kinetic performance analysis of different zwitterionic hydrophilic interaction liquid chromatography polymer columns was performed, wherein two different monolithic materials were compared with a particulate material. These materials were already studied in previous work, where it was demonstrated that they all display very low diffusion coefficients in the mesoporous zone. To further unravel the consequences of this low mesoporous zone diffusion, several typical column performance analysis approaches were considered. 

This consisted of first assessing the structure and porosity of the different packings via SEM and ISEC. The obtained SEM pictures revealed a relatively large heterogeneity for the monolithic columns, especially for the throughpores. The particle packed column on the other hand, consisted of spherical particles with a rather large particle size distribution. The ISEC measurements resulted in external porosity values that were in line with those expected for monolithic columns, but were relatively high for the particle packed column, especially considering their large particle size distribution. Therefore, a value of ε_e_ = 0.34 was considered for further calculations, based on literature data for packings with similar particle size distributions.

Next, plate heights were measured over a broad range of velocities on all columns for compounds with similar retention factors. The obtained plate heights were fitted to a plate height equation, and the obtained fitting parameters (A, B and C-terms) compared. The obtained high A-term values for the ZIC-pHILIC column suggested a high heterogeneity of the packed particle column, while the C-term values were rather similar for all columns. Further inspection of the plate height curves of the ZIC-pHILIC column, however, revealed an amount of perfusion through the particles that impacted the fitted A- and C-term values and made them useless for further comparison.

In an attempt to compare the performance of the different column formats via reduced plate height curves, the requirement for a single, representative characteristic length for each packing was complicated by their largely differing structure and the heterogeneity observed for their structural elements. Interestingly, the (reduced) plate height curves revealed a rather random pattern in the high-velocity region of the curves, where no clear correlation was observed with k″, suggesting other parameters in the general plate height model could be responsible for this observation. Further investigation of the plate height curves of the monolithic columns, where the high-velocity zone of the plate height curve was considered c_s_-term dominated, revealed a correlation between the obstruction factor in the mesoporous zone and the molecular weight of the compounds. Although speculative at this instance, this suggests that a compound experiences more obstruction against free movement in the mesopores of these polymer monoliths as its MW increases. 

To overcome the problems encountered with the traditional column performance comparison methods (reduced plate height plots; A-, B-, C-constant analysis), kinetic plots were constructed to compare the different column formats in a geometry-independent way. This was done for a compound (uracil) that had a similar k″ on all materials. These revealed that the monoliths—despite their observed heterogeneity—performed better than the ZIC-pHILIC column evaluated in this study, over the entire relevant range of plate counts. This was attributed on the one hand to their smaller skeleton and domain sizes that led to higher efficiencies, and on the other hand, to their high permeability, resulting from to their high external porosity and the occurrence of occasional preferential flow paths due to the presence of some very large macropores. This study clearly demonstrates the utility of the kinetic plot method to compare the kinetic performance of different column formats, that can be difficult to characterize in terms of geometric dimensions. As a follow-up, analysts could also compare column supports in terms of other parameters, such as the ‘greenness’ of their corresponding analytical procedures, using for example the Analytical GREEnness Metric Approach [35].

## Figures and Tables

**Table 1:** Structural characteristics (d_glob_, d_tp_, d_dom_ and d_Halasz_) and column porosities of the columns evaluated in this work. The reported sizes show the average values of at least 100 independent measurements and their standard deviations.

Column	$d_{skel}$ (µm)	$d_{tp}$ (µm)	$d_{p}$ or $d_{dom}$ (µm)	d_Halasz_ (µm)	$\varepsilon_{e}$	$\varepsilon_{T}$	$\varepsilon_{i}$
poly(SPE-co-EDMA)	0.7 ± 0.1	0.8 ± 0.4	1.5 ± 0.4	5.2	0.6923	0.7830	0.2948
poly(SPE-co-MBA)	1.7 ± 0.3	1.6 ± 0.7	3.3 ± 0.8	5.7	0.6995	0.7370	0.1248
ZIC-pHILIC	/	/	4.7 ± 1.1	5.5	0.4398	0.6040	0.2931

**Table 2:** A-, B- and C-term values obtained by fitting the experimental plate height data to Equation (10), either fitting all terms freely (BFIT) or fixing the B-term value to the value obtained by peak parking in [21], and subsequently fitting A and B (BPP).

Column	Compound	k″	H_min_ (mm)	Equation (10) B_FIT_			Equation (10) B_PP_		
				A (mm^1/2^/s^1/2^)	B (mm²/s)	C (s)	A (mm^1/2^/s^1/2^)	B (mm²/s)	C (s)
SPE-co-EDMA	Uracil	1.92	2.17 × 10^−2^	1.38 × 10^−2^	3.35 × 10^−3^	1.22 × 10^−2^	1.25 × 10^−2^	3.48 × 10^−3^	1.30 × 10^−2^
	Uracil	3.08	2.76 × 10^−2^	2.29 × 10^−2^	3.80 × 10^−3^	1.31 × 10^−2^	2.89 × 10^−2^	3.27 × 10^−3^	8.74 × 10^−3^
	Thiourea	4.96	1.37 × 10^−2^	6.90 × 10^−3^	3.83 × 10^−3^	3.71 × 10^−3^	7.59 × 10^−3^	3.70 × 10^−3^	3.21 × 10^−3^
	Thiourea	7.00	1.53 × 10^−2^	8.43 × 10^−3^	4.11 × 10^−3^	4.11 × 10^−3^	9.53 × 10^−3^	3.91 × 10^−3^	3.42 × 10^−3^
	Hypoxanthine	10.29	2.47 × 10^−2^	1.46 × 10^−2^	3.53 × 10^−3^	2.18 × 10^−2^	1.39 × 10^−2^	3.64 × 10^−3^	2.23 × 10^−2^
SPE-co-MBA	Uracil	1.86	1.70 × 10^−2^	1.78 × 10^−2^	2.08 × 10^−3^	1.38 × 10^−3^	1.56 × 10^−2^	2.31 × 10^−3^	2.80 × 10^−3^
	Adenosine	2.93	2.12 × 10^−2^	2.22 × 10^−2^	1.53 × 10^−3^	1.96 × 10^−2^	1.96 × 10^−2^	1.80 × 10^−3^	2.11 × 10^−2^
	Thiourea	4.73	1.22 × 10^−2^	8.80 × 10^−3^	2.66 × 10^−3^	1.63 × 10^−3^	6.25 × 10^−3^	3.05 × 10^−3^	3.20 × 10^−3^
	Uridine	6.37	1.48 × 10^−2^	1.25 × 10^−2^	1.47 × 10^−3^	1.21 × 10^−2^	1.21 × 10^−2^	1.72 × 10^−3^	1.20 × 10^−2^
	Inosine	9.69	1.36 × 10^−2^	1.42 × 10^−2^	1.30 × 10^−3^	5.98 × 10^−3^	1.41 × 10^−2^	1.25 × 10^−3^	6.04 × 10^−3^
ZIC-pHILIC	Uracil	2.18	2.78 × 10^−2^	3.84 × 10^−2^	2.07 × 10^−3^	0.00	3.92 × 10^−2^	1.84 × 10^−3^	0.00
	Adenosine	3.70	4.11 × 10^−2^	8.70 × 10^−2^	1.37 × 10^−3^	9.12 × 10^−3^	8.31 × 10^−2^	1.50 × 10^−3^	1.26 × 10^−2^
	Uridine	6.39	2.87 × 10^−2^	4.98 × 10^−2^	1.29 × 10^−3^	5.71 × 10^−3^	4.42 × 10^−2^	1.61 × 10^−3^	1.05 × 10^−2^
	Uridine	7.31	3.26 × 10^−2^	5.45 × 10^−2^	1.50 × 10^−3^	9.31 × 10^−3^	5.84 × 10^−2^	1.25 × 10^−3^	5.97 × 10^−3^
	Uridine	10.54	3.14 × 10^−2^	4.71 × 10^−2^	1.62 × 10^−3^	1.31 × 10^−2^	5.33 × 10^−2^	1.33 × 10^−3^	7.41 × 10^−3^

## Data Availability

Data available upon written request to the corresponding author.

## Supplementary Materials

The following supporting information can be downloaded at: https://www.mdpi.com/article/10.3390/molecules28072902/s1, Table S1: Column dimensions and chromatographic conditions used to evaluate the performance of the columns; Figure S1: Column pressure as a function of the linear velocity for the three columns evaluated in this work; Figure S2: Reduced plate height curves of h versus ν_i_ obtained by using the domain size (d_dom_) as the characteristic length for the monolithic columns, and the particle size (d_p_) for the particle packed column.
