# Supplementary material for: In-Depth Performance Analysis and Comparison of Monolithic and Particulate Zwitterionic Hydrophilic Interaction Liquid Chromatography Polymer Columns
Source: Molecules. 2023 Mar 23;28(7):2902. doi: 10.3390/molecules28072902 (PMC10095884; doi:10.3390/molecules28072902)
Supplement: Supplementary file 1 [file molecules-28-02902-s001.zip › molecules-2279821-supplementary.pdf]

Supporting Information

In-depth performance analysis and comparison of monolithic and particulate zwitterionic hydrophilic interaction liquid chromatography polymer columns

Haibin Li[1,2], Zhengjin Jiang[2], Gert Desmet[3], Deirdre Cabooter[1]*

[1] University of Leuven (KU Leuven), Department for Pharmaceutical and Pharmacological Sciences, Pharmaceutical Analysis, Herestraat 49, Leuven, Belgium

[2] Institute of Pharmaceutical Analysis, College of Pharmacy, Jinan University, Guangzhou 510632, China

[3] Vrije Universiteit Brussel, Department of Chemical Engineering, Pleinlaan 2, 1050 Brussel, Belgium

(*) corresponding author:

tel.: (+) 32 (0)16.32.34.42, fax: (+) 32 (0)16.32.34.48,

E-mail: deirdre.cabooter@kuleuven.be (D. Cabooter)

Table S-1: Column dimensions and chromatographic conditions used to evaluate the performance of the columns. The measured molecular diffusion coefficients (D_m) and molecular weight (MW) are also shown. Table adapted from [20]

Column	Dimensions (mm)	Mobile phase ACN/H_2O (v/v) (3 mM NH_4Ac total, pH 6.0)	Compound	k''	D_m ($\times 10^{-9}$ m^2/s)	MW (g/mol)
poly(SPE-co-EDMA) monolithic column	0.1 × 234	95/5	Uracil	1.92	1.99	112
		96.5/3.5	Uracil	3.08	2.11	112
		95.5/4.5	Thiourea	4.96	2.42	76
		96.5/3.5	Thiourea	7.00	2.49	76
		96.5/3.5	Hypoxanthine	10.29	1.85	136
poly(SPE-co-MBA) monolithic column	0.1 × 226	93/7	Uracil	1.86	1.87	112
		93/7	Adenosine	2.93	1.29	267
		93/7	Thiourea	4.73	2.36	76
		93/7	Uridine	6.37	1.34	244
		92/8	Inosine	9.69	1.21	268
ZIC-pHILIC	2.1 × 150	89/11	Uracil	2.18	1.73	112
		89/11	Adenosine	3.70	1.23	267
		90.5/9.5	Uridine	6.39	1.32	244
		91.5/8.5	Uridine	7.31	1.33	244
		93/7	Uridine	10.54	1.34	244

Figure S-1: Column pressure as a function of the linear velocity for the three columns evaluated in this work: (●) poly(SPE-co-EDMA) monolithic column, (◆) poly(SPE-co-MBA) monolithic column, (■) ZIC-pHILIC column. The dashed lines indicate the linear trend lines, fitting equations and R^2-values are also shown.

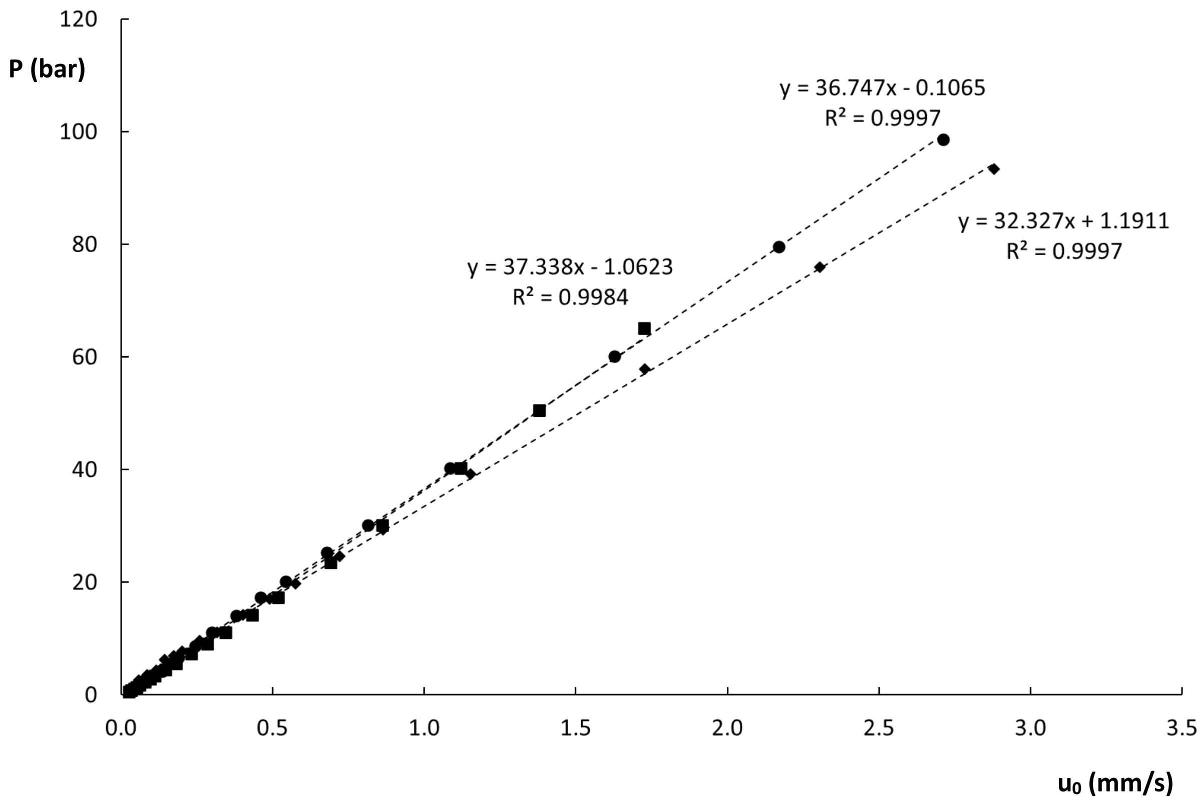

Figure S-2: Reduced plate height curves of h versus ν_i obtained by using the domain size (d_{dom}) as the characteristic length for the monolithic columns, and the particle size (d_p) for the particle packed column. Curves for the a) poly(SPE-co-EDMA) monolithic column, b) poly(SPE-co-MBA) monolithic column and c) ZIC-pHILIC column. Mobile-phase conditions as in Table 1.

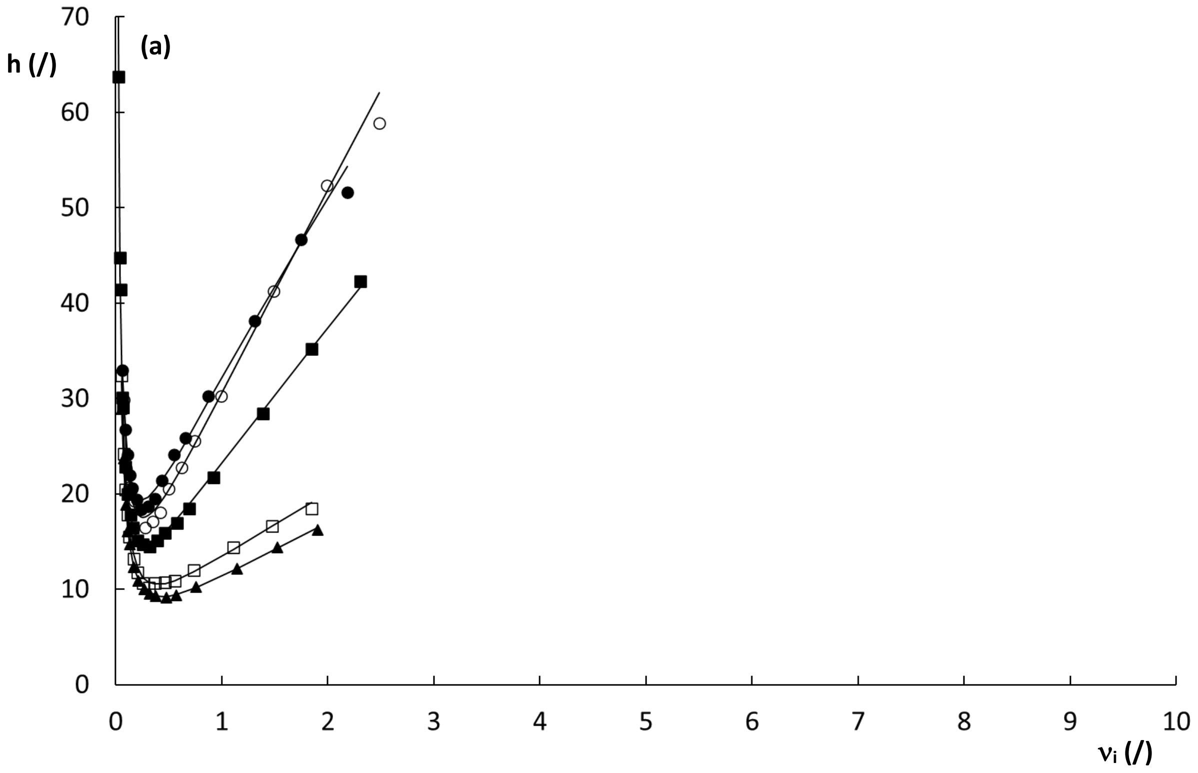

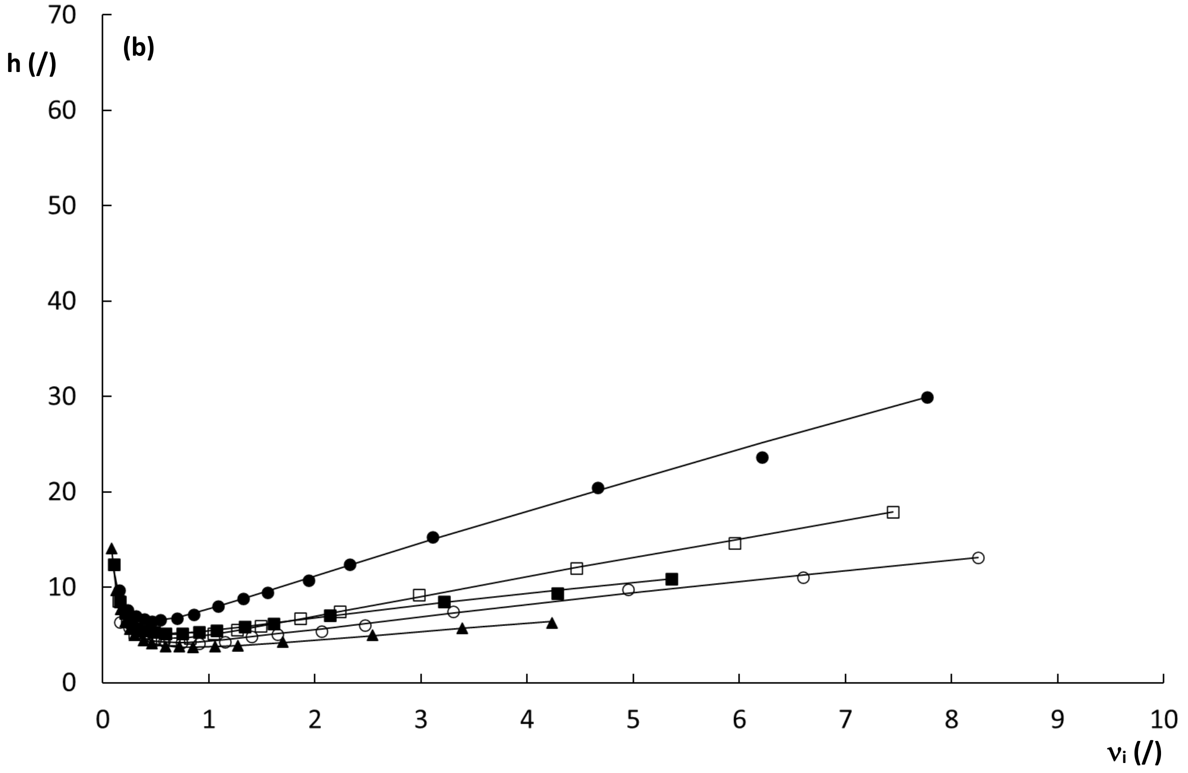

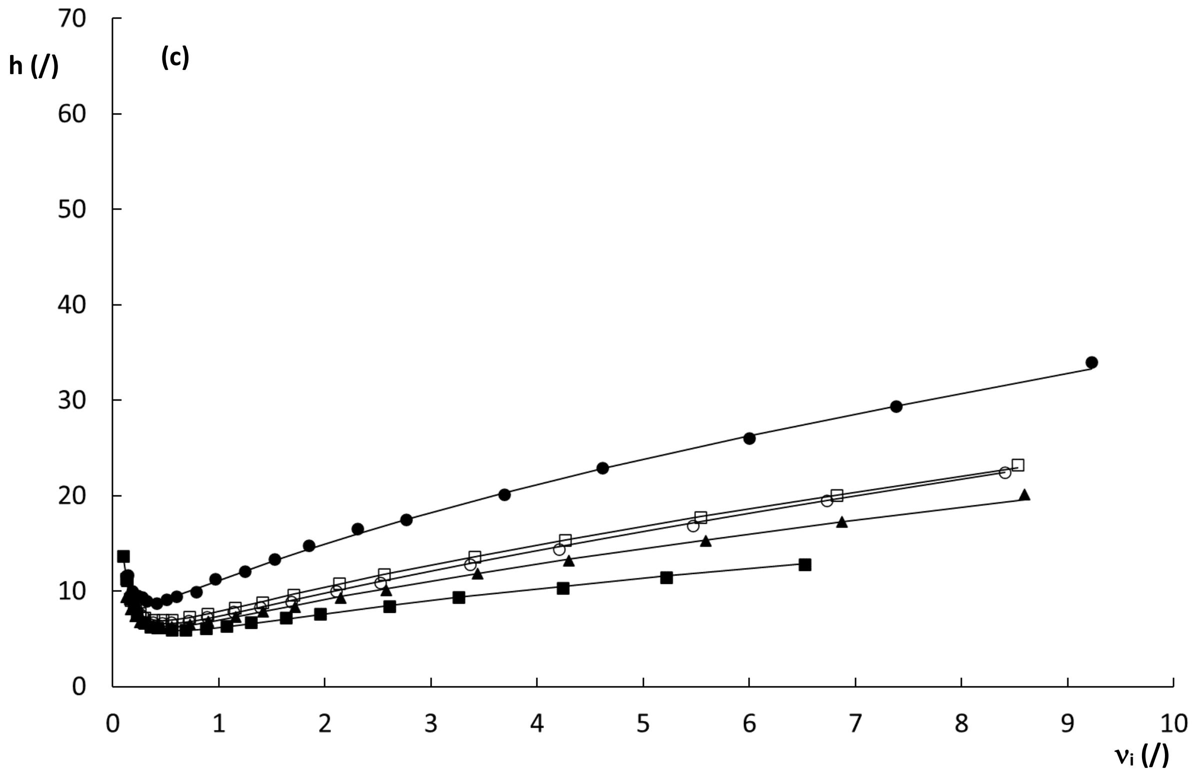

h (/)
(c)
70
60
50
40
30
20
10
0
0
1
2
3
4
5
6
7
8
9
10
ν_i (/)
